# Sixty-four or four-and-sixty? The influence of language and working memory on children's number transcoding

**DOI:** 10.3389/fpsyg.2014.00313

**Published:** 2014-04-11

**Authors:** Ineke Imbo, Charlotte Vanden Bulcke, Jolien De Brauwer, Wim Fias

**Affiliations:** ^1^Department of Experimental Psychology, Ghent UniversityGhent, Belgium; ^2^Department of Experimental-Clinical and Health Psychology, Ghent UniversityGhent, Belgium; ^3^Code, Expertise Centre for Development and Learning and Department of Applied Psychology, Thomas More University CollegeAntwerp, Belgium

**Keywords:** number transcoding, number language, working memory, place-value understanding, transcoding errors, inversion errors

## Abstract

Number transcoding (e.g., writing 64 when hearing “*sixty-four*”) is a basic numerical skill; rather faultlessly performed in adults, but difficult for children. In the present study, children speaking Dutch (an inversed number language) and French (a non-inversed number language) wrote Arabic digits to dictation. We also tested their IQ and their phonological, visuospatial, and executive working memory. Although the number of transcoding errors (e.g., hearing 46 but writing 56) was equal in both groups, the number of inversion errors (e.g., hearing 46 but writing 64) was significantly higher in Dutch-speaking than in French-speaking children. Regression analyses confirmed that language was the only significant predictor of inversion errors. Working-memory components, in contrast, were the only significant predictors of transcoding errors. Executive resources were important in all children. Less-skilled transcoders also differed from more-skilled transcoders in that they used semantic rather than asemantic transcoding routes. Given the observed relation between number transcoding and mathematics grades, current findings may provide useful information for educational and clinical settings.

## Introduction

Numeracy is extremely important in our everyday life (e.g., banking, cooking, shopping) and it gets even more important given the challenges of our modern society (e.g., population control, stock market crashes, climate change, and health risks, e.g., Reyna and Brainerd, 2007). Given the crucial role of numeracy, researchers started to search for its building blocks. The present study focuses on one of these building blocks, number transcoding or the ability to translate between number formats (verbal to Arabic or Arabic to verbal).

### Number transcoding

Numbers come in various formats, such as Arabic digits (e.g., 46) and number words (e.g., “*forty-six*”). Number transcoding refers to the process in which a number is “translated” from one format into another one. Examples are writing down a dictated number or saying aloud an Arabic digit. Although these tasks are usually faultlessly performed by adults, they pose significant problems to young children. Throughout development, children learn to map the early-learned number words onto the respective Arabic symbols, increasing the overlap between the different formats (Kucian and Kaufmann, 2009).

The Arabic number system is rather simple, as it consists of only 10 elements (0, 1, 2, 3, 4, 5, 6, 7, 8, and 9) and one principle (i.e., the place value principle, according to which the value of a digit increases by a power of 10 with each step to the left). Verbal number systems, in contrast, are much more complicated. They rely on a limited lexicon, organized in different lexical classes such as units (“*one*” to “*nine*”), decades (“*ten*” to “*ninety*”), hundreds, and thousands. Most lexicons also entail particulars such as “*eleven*” and “*twelve*.” Because only a few quantities can be designated by a single word, a syntax provides the rules for making larger word sequences. Examples are additive rules (e.g., “*one hundred and sixty*” → 100 + 60 = 160) and multiplicative rules (e.g., “*four hundred*” → 4 × 100 = 400). Errors against these rules would result in 10060 and 4100, respectively.

Cognitive research into number transcoding was rather scarce, but gained renewed attention recently. The most widely used task to study number transcoding is writing Arabic numbers to dictation. Using this task, it has been shown that error rates are high, but decline across development: 49–54% in 6-year-olds, 22–54% in 7-year-olds, and 16–36% in 8-year-olds (Power and Dal Martello, 1990; Noël and Turconi, 1999; Camos, 2008; Zuber et al., 2009; Krinzinger et al., 2011; Pixner et al., 2011; Simmons et al., 2011). Syntactic errors, in which the number's elements are correct but its magnitude is not (e.g., “*one hundred twenty-three*” → 10023) are generally more frequent in children's performance than are lexical errors, in which a number's elements are incorrect (e.g., “*one hundred twenty-three*” → 124) (Seron et al., 1992; Sullivan et al., 1996).

Cognitive models of number transcoding offer a useful framework to understand and investigate number transcoding. A first category consists of *semantic* models, according to which the number word is first transformed into a semantic magnitude representation, and then into its constituent Arabic digits (e.g., McCloskey et al., 1985; Power and Dal Martello, 1990; McCloskey, 1992). Semantic models predict that number transcoding will be harder for larger numbers.

A second category consists of *asemantic* models, according to which number transcoding does not require any semantic magnitude representation (e.g., Deloche and Seron, 1987; Barrouillet et al., 2004). The ADAPT (A Developmental Asemantic Procedural Transcoding) model of Barrouillet et al. (2004), for example, consists of representational units in a mental lexicon, and production rules, according to which these units are combined. Since each part of the verbal number word is sent to working memory for storage and processing, the ADAPT model not only predicts that attentional resources are crucial in number transcoding, but also that number transcoding will be harder as the frequency of digits in the number increases.

A third category of models are connectionist models, which do not rely on rules or operators. The connectionist transcoding model of Verguts and Fias (2006) simulates transcoding on the basis of number frequency-based learning algorithms. Developed in the context of Arabic to verbal transcoding, the model generally demonstrates that a model without prior knowledge learns to read aloud numbers by developing two routes: a lexical route and a syntactic route. The lexical route is a direct route that maps Arabic input to phonological output representations without any intermediate steps. The syntactic route is an indirect route that applies principles of syntax to convert Arabic input to output phonology. These two routes do not involve any number magnitude representations, making the model essentially a non-semantic one. Yet, although not explicitly included in the model, Verguts and Fias (2006) do not exclude the possibility that an additional semantic route might exist for reading aloud a specific and restricted type of numbers, namely very small numbers and numbers with a very specific semantic meaning (like 1939). Their high frequency of occurrence (given their numerical magnitude, Dehaene and Mehler, 1992) and/or their special semantic status may make their semantic meaning more salient compared to other numbers with comparable numerical magnitude.

Whether children take a semantic or asemantic route depends on their numerical skill, as shown by Van Loosbroek et al. (2009). Nine-years old children with arithmetical disabilities needed more planning time writing large 1-digit numbers (e.g., 8) than when writing small 1-digit numbers (e.g., 3), suggesting the use of a semantic route. Control children, in contrast, did not show such a problem size effect for 1-digit numbers, suggesting an asemantic route. For 2- and 3-digit numbers, both groups of children showed a problem size effect, but the effect was smaller for control children.

### Language

Interestingly, recent research suggests that children's number transcoding is influenced by language. Writing numbers to dictation would be especially difficult for children speaking an inversed number language, such as Dutch or German, where the pronunciation of two-digit numbers is inversed (e.g., 64 is pronounced as “*four-and-sixty*”). In a cross-cultural transcoding study, Nuerk et al. (2005) observed that 7-year-old Japanese-speaking children made about six times fewer transcoding errors than did their German-speaking counterparts, and about eight times fewer inversion errors (e.g., hearing “*four-and-sixty*” but writing 46). Similarly, Krinzinger et al. (2011) showed that Dutch- and German-speaking children made more transcoding errors than did French-speaking children. Pixner et al. (2011), finally, showed that 7-year-old children speaking Czech (which has an inverted and a non-inverted number language)^1^ made 49% errors when numbers were dictated in the inverted number language, of which about half were inversion-related. When numbers were dictated in the non-inverted number language, errors dropped down to 37%.

One goal of the present study was to shed more light on these language effects in children's number transcoding. To that end, we tested children speaking an inversed number language (Dutch) and children speaking a non-inversed number language (French)^2^. Importantly, both groups were Belgian (Flemish and Walloons, respectively), so that cross-cultural differences in educational systems and math curricula were minimized. If the inversion property of the Dutch number language really affects children's number transcoding, more inversion errors should occur in the Dutch-speaking than in the French-speaking children. As neither semantic nor asemantic transcoding models do in their current form account for inversion errors, observing a reasonable number of inversion errors would urge for a revision of the transcoding models currently available.

However, it is interesting to examine not only the errors related to one specificity of a number language (such as inversion), but also the errors *not* related to this specificity. In this context, two accounts can be proposed (cf. Pixner et al., 2011). One possibility is that a complex number language puts a higher burden on children's available resources than a less complex number language. The complexity of an inverted number language would then also influence the execution of other (more general) transcoding rules, with more non-inversion errors in Dutch-speaking than in French-speaking children. In contrast, if the complexity of an inverted number language does not influence the execution of other transcoding rules, we would expect an similar number of non-inversion errors in Dutch-and French-speaking children.

### Working memory

Another goal of our study was to test the role of working memory in children's number transcoding. Camos (2008) showed that French 8-year-olds with lower working-memory spans made more transcoding errors than did 8-year-olds with higher working-memory spans. However, since Camos used a general working-memory span task (counting dots), it was impossible to pinpoint which working-memory component (phonological, visuospatial, or executive, following the three-component model of working memory; Baddeley and Hitch, 1974; Baddeley, 1992) was most important in children's number transcoding. Three recent studies (Zuber et al., 2009; Pixner et al., 2011; Simmons et al., 2011) extended Camos' (2008) research by including phonological, visuospatial, and executive tasks. As such, they could distinguish the impact of the different working-memory codes in number transcoding.

Zuber et al. (2009) showed that executive working memory was the strongest predictor of transcoding performance in German-speaking first graders: the higher a child scored on executive working-memory tasks, the fewer transcoding errors it committed. Further analyses showed that executive working memory was predictive of inversion-related errors whereas visuospatial working memory was predictive of non-inversion-related errors. The important role of the central executive was confirmed by Pixner et al. (2011). They showed that executive working memory predicted first graders' error rates in both inverted and non-inverted Czech number languages. Somewhat different results were obtained by Simmons et al. (2011). In their study on first and third graders speaking a non-inversed language (English), visuospatial working memory was the only significant predictor of transcoding performance. In contrast to Simmons et al.'s expectations, executive working memory was not predictive. Phonological working memory was predictive in none of the above-mentioned studies (Zuber et al., 2009; Pixner et al., 2011; Simmons et al., 2011), which is surprising because transcoding is commonly assumed to index verbal number processing.

It is, however, important to note that neither study tested the role of working memory across languages. Camos (2008) only tested French-speaking children, Zuber et al. (2009) only tested German-speaking children, Pixner et al. (2011) only tested Czech-speaking children, and Simmons et al. (2011) only tested English-speaking children. Although the results of these studies seem to suggest that executive working memory is more important in inverted number languages (Czech and German) than in non-inverted number languages (English), this conclusion might be premature. Indeed, the studies do not only differ in the languages they tested and in the tasks they used, but also in cultural and educational practices. In the present study, we tested both Dutch- and French-speaking children, which allowed us to test the role of working memory in inversed and non-inversed number languages, relatively independent of cultural and educational differences. If transcoding requires working-memory resources, working memory should predict both Dutch- and French-speaking children's transcoding performance. If transcoding is more resource-demanding in inversed than in non-inversed languages (Camos, 2008; Zuber et al., 2009; Pixner et al., 2011; Simmons et al., 2011), working memory should be more predictive in Dutch-speaking than in French-speaking children. However, if executing the inversion rule is what makes transcoding difficult, working memory should specifically predict Dutch children's number of inversion errors.

Another reason why Zuber et al. (2009) and Pixner et al. (2011) observed a role for executive working memory whereas Simmons et al. (2011) did not, may involve age. Zuber et al. (2009) and Pixner et al. (2011) tested first graders, who have no formal experience with numbers larger than 20, whereas Simmons and colleagues tested first and third graders, the latter having lots of formal experience with numbers larger than 20.

### The present study

In sum, we wanted to test several hypotheses. First, which working-memory components are important in number transcoding? Second, what is the role of language in number transcoding? Do children speaking an inversed number language really make more transcoding errors than do children speaking a non-inversed number language? And if so, do children speaking an inversed number language rely more heavily on their working memory? Third, is the differentiation between semantic and asemantic transcoding routes (as observed by Van Loosbroek et al., 2009) also present in typically developing children?

Based on a pretest, we decided to focus on second graders, because first graders did not show enough knowledge of transcoding rules and because third graders did not make enough errors to allow a meaningful interpretation. Of the second graders we selected the 10 less- and more-skilled transcoders in each language group, which were further tested on IQ and working memory. Analyses on the percentages of transcoding and inversion errors are conducted, as well as analyses concerning the role of working memory. By dividing the children in less- and more-skilled transcoders, we were able to test whether the differentiation between semantic and asemantic transcoding routes (cf. Van Loosbroek et al., 2009) is also present in typically developing children. Since the asemantic route can be seen as developmentally more advanced than the semantic one (because there is no problem-size effect, the asemantic route can process more numbers in less time), we predicted that more-skilled children would use the asemantic route while less-skilled children would rather use the semantic route.

## Methods

### Participants

A total of 87 children participated: 49 Dutch-speaking second graders (22 girls; mean age: 7 years 7 months) attending a school in the Flemish part of Belgium and 38 French-speaking second graders (20 girls; mean age: 7 years 7 months) attending a school in the Walloon part of Belgium. Mean age did not differ between both groups, *t*_(38)_ = 0.00; *p* = 1.00. Children only participated if they and their parents consented. None of the children presented sensory or motor deficiencies or any psychiatric diagnosis. The children received a small reward after participation. In each language group, children were ranked based on the total number of transcoding errors in number dictation. Ten children with the fewest and most errors were selected as the more-skilled and less-skilled transcoders group, respectively (*M*_*transcoding errors*_ = 35.7 for less-skilled Dutch-speaking children, *M*_*transcoding errors*_ = 32.7 for less-skilled French-speaking children, *M*_*transcoding errors*_ = 0.30 for more-skilled Dutch-speaking children, *M*_*transcoding errors*_ = 0.7 for more-skilled French-speaking children). None of these children were bilingual.

### Materials and procedure

The number dictation task was presented to all children (*n* = 87), see below for a description. IQ and working memory were tested in the selected group only (*n* = 40), on two different days. On the first day, working memory was tested by means of two phonological tasks (Digit and Letter span forward), two visuospatial tasks (Corsi blocks forward and Mazes memory), and four executive tasks (Digit and Letter span backward, Corsi blocks backward, and Sun moon Stroop). The Digit span, Corsi blocks, and Mazes memory tasks were taken from the Working Memory Test Battery for Children (WMTB-C, Pickering and Gathercole, 2001). On the second day, IQ was tested by means of two verbal subtests (Similarities and Vocabulary) and two performance subtests (Block design and Picture arrangement) of the WISC-III (Wechsler, 2002). These subtests provide a valid estimation of children's total IQ (Grégoire, 2001). Test-retest reliability is 0.92. The working-memory and IQ test series took about half an hour per child. If available, information about reliability of the measures is provided.

#### Number dictation task

The item set consisted of five 1-digit numbers, twenty 2-digit numbers, and forty 3-digit numbers. We made sure that each category of the ADAPT model was represented (see Supplementary material). The group-administered dictation took about 20 min and was conducted by the same, bilingual experimenter in both schools. The children received a booklet with 65 small pictures and were asked to write down the dictated number near the picture, mentioned as well during dictation (e.g., “write twenty-four next to the sun”). The pictures were used to motivate the children and to structure the responses of the dictation task. Each number was read aloud twice. When children did not know how to write the number, they were told to put an “X” instead.

#### Digit span forward and backward

The experimenter read a series of single-digit numbers at a rate of one digit per second, beginning with a string of 2 digits and proceeding to progressively larger strings, with a maximum of 9 digits. The child was required to repeat the exact sequence in the same (resp. reversed) order. There were six strings for each length, and testing was stopped when the child missed three sequences of the same length. Performance was scored as the number of correctly repeated digit strings. Test-retest reliability is 0.81 for digit span forward and 0.62 for digit span backward (WMTB-C, Pickering and Gathercole, 2001).

#### Letter span forward and backward

The method of this task is similar to the digit span, but the stimuli were letters instead of digits. Vowels and the letter w (“*double v*” in French) were not included, and all series consisted of phonologically different letters (cf. Butterworth et al., 1996). Performance was scored as the number of correctly repeated letter strings.

#### Corsi blocks forward and backward

The children were presented with nine identical wooden blocks in random positions on a wooden board. The children were told that these blocks were “stones in a pond.” Using a plastic duck, the experimenter tapped on a sequence of blocks at the rate of one block per second, beginning with a 2-block sequence and proceeding to progressively larger sequences, with a maximum of 9 blocks. The child was asked to reproduce the exact sequence in the same (resp. reversed) order. There were six sequences for each length, and testing was stopped when the child missed three sequences of the same length. Performance was scored as the number of correctly repeated sequences. Test-retest reliability for Corsi blocks forward is 0.53 (WMTB-C, Pickering and Gathercole, 2001).

#### Mazes memory

The child is presented with a picture of a maze, and a picture of an identical maze with the correct path drawn on it. The picture is removed, and the child's task was to duplicate the path in the response booklet. The difficulty level of the mazes started at span 2 (which corresponds to two walls in the maze), and proceeded to progressively larger spans, with a maximum of 8. At each level, the mazes get larger by one wall. There were six mazes for each level, and testing was stopped when the child missed three sequences of the same level. Performance was scored as the number of correctly drawn mazes.

#### Sun moon stroop

This variant of the Stroop task is composed of two pages containing rows of pictures of suns and moons arranged pseudo-randomly (Archibald and Kerns, 1999). In the first condition, children are asked to say “sun” for a picture of a sun and “moon” for a picture of a moon. In the second condition, children were asked to say “sun” for a picture of a moon and “moon” for a picture of a sun. In both conditions, children were instructed to go as quickly and accurately as possible, within a time limit of 45 s. They had to stop and correct any errors that were made. If a child reached the end before the 45 s had elapsed, the time required to complete the page was recorded and the number that would have been correct within the time limit was estimated. A performance score was calculated by subtracting the number of correct responses in the first condition from the number of correct responses in the second condition and then dividing this difference by the number of correct responses in the first condition. Test-retest reliability is 0.86 (WMTB-C, Pickering and Gathercole, 2001).

## Results

### Error analyses

**Figure 1:**
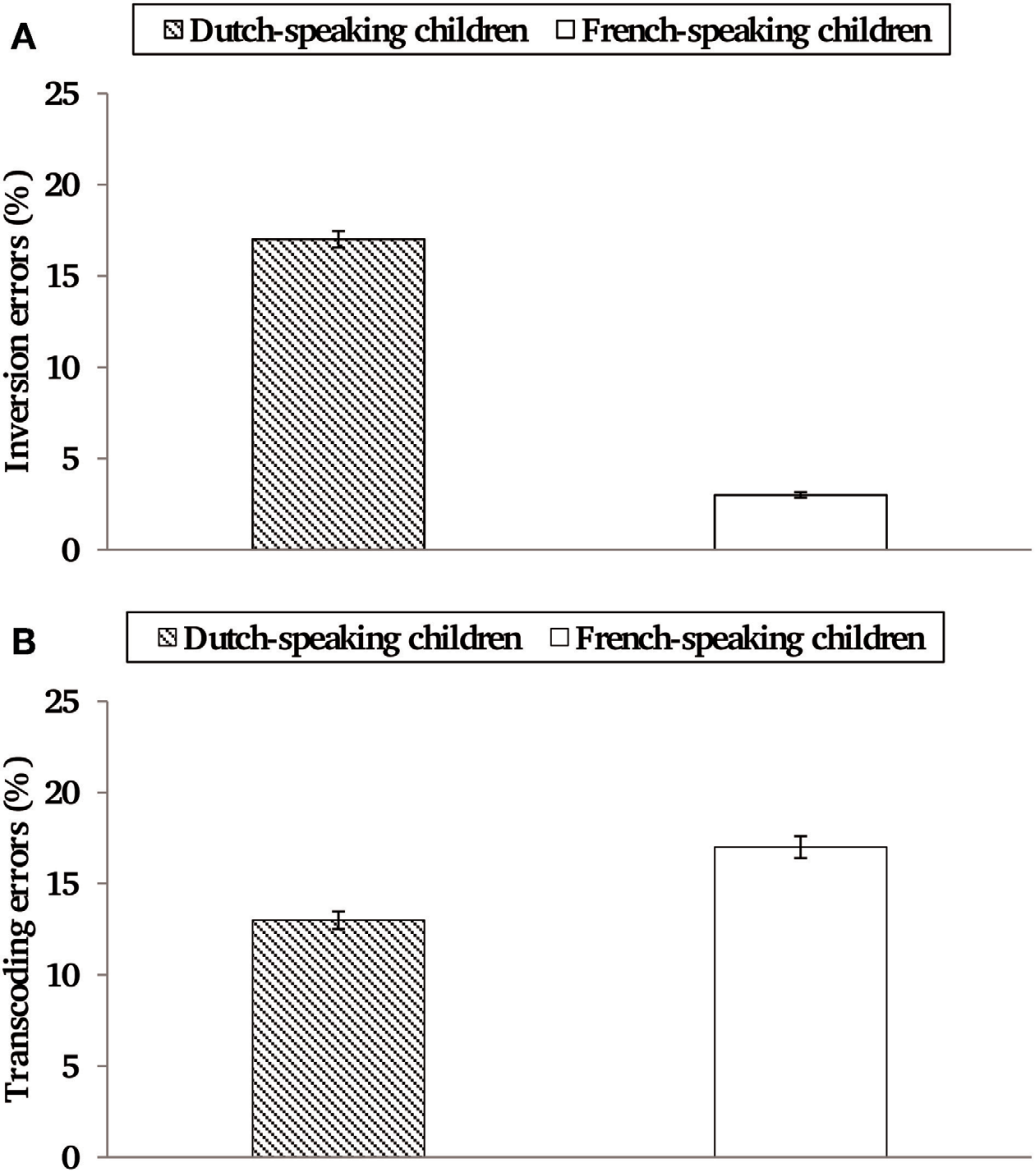
**Percentage of transcoding errors (A) and inversion errors (among the transcoding errors) (B) as a function of Language.** Standard deviations were given for each group.

A categorization of all errors can be found in Table 1. Dutch- and French-speaking children made an equal number of transcoding errors [13% vs. 17%, *t*_(85)_ = −1.05, *p* = 0.30], see Figure 1. The percentage of transcoding errors was significantly higher on 3-digit than on 2-digit numbers, for both Dutch- and French-speaking children, *t*_(96)_ = −4.37 (*p* < 0.001) and *t*_(74)_ = −4.53 (*p* < 0.001), respectively. The percentage of inversions errors (among the total number of errors) was higher in Dutch-speaking than in French-speaking children (17 vs. 3%), *t*_(85)_ = 3.56 (*p* < 0.001), see Figure 1.

**Table 1:** **Items that were left open and erroneously transcoded as a function of Language (percentages between brackets)**.

	Dutch-speaking	French-speaking
Left open	162 ± 7 (5)	154 ± 8(6)
Lexical error^a^	36 ± 1 (1)	52 ± 2(2)
Syntactic error^b^	293 ± 10 (9)	192 ± 10(8)
Combined error^c^	90 ± 5 (3)	189 ± 9(8)

a Lexical error = when a lexical element is substituted by another one (e.g., 25 → 24).

b Syntactic error = when the elements of the number are correct but its magnitude is not (e.g., 123 → 10023).

c Combined error = when both lexical and syntactic rules are violated (e.g., 467 → 40057).

### IQ and working memory

Based on the number of transcoding errors, we selected the 10 more-skilled and 10 less-skilled transcoders in each language group. These children's IQ and working memory was further tested. Importantly, IQ scores differed neither between less- (*M* = 106) and more-skilled (*M* = 112) transcoders nor between Dutch- (*M* = 106) and French-speaking children (*M* = 110) [respectively *t*_(38)_ = −1.99, *p* = 0.06; *t*_(38)_ = −1.06, *p* = 0.29]. The less- and more skilled transcoders differed in working-memory scores though. As can be seen in Table 2, the less-skilled transcoders scored lower on the Digit span forward, *t*_(38)_ = 2.40 (*p* < 0.05) and on the Letter span backward, *t*_(38)_ = 3.39 (*p* < 0.01). Correlations between IQ, age, and the working-memory tasks can be found in Table 3. To explore which working-memory components play a unique role in children's number transcoding, regression analyses were performed.

**Table 2:** **Scores on the working-memory tasks for more- and less-skilled transcoders**.

	**Phonological**		**Visuospatial**		**Executive**			
	**Digit forward**	**Letter forward**	**Corsi blocks**	**Mazes Memory**	**Digit backward**	**Letter backward**	**Corsi backward**	**Sun moon**
More-skilled	27	23	19	13	12	11	14	14
Less-skilled	24	21	20	10	10	7	12	−11
*t*-test	−2.4^*^	1.78	0.46	1.29	−1.54	−3.39^**^	−1.23	1.24

* p < 0.05,

** p < 0.01, (n = 20).

**Table 3:** **Correlations between IQ, age, and the working-memory tasks**.

	**1**	**2**	**3**	**4**	**5**	**6**	**7**	**8**	**9**	**10**
1. IQ		−0.12	0.45^**^	0.22	0.24	0.11	−0.01	0.34^*^	0.15	0.10
2. Age			0.21	0.22	0.02	0.19	−0.16	0.05	0.16	0.04
3. Digit forward				0.38^*^	0.23	0.10	0.35^*^	0.55^**^	0.27	0.13
4. Letter forward					0.06	−0.10	0.07	0.03	0.01	−0.10
5. Corsi forward						0.02	0.18	0.14	0.29	0.22
6. Mazes memory							0.20	0.12	0.23	−0.06
7. Digit backward								0.64^**^	0.24	0.25
8. Letter backward									0.35^*^	0.21
9. Corsi backward										0.11
10. Sun moon										

* p < 0.05,

** p < 0.01, (n = 40).

### Regression analyses

The regression analyses incorporated compound scores for each working-memory component (phonological, visuospatial, and executive). These compound scores were calculated as the mean of the respective *z*-scores (for a similar procedure, see Barrouillet et al., 2008; Zuber et al., 2009; Pixner et al., 2011). Language (Dutch vs. French) was also included as a predictor, as were the interactions between language and the three working-memory components. Finally, IQ and age were included to ensure that potential working-memory influences were not due to IQ- or age-related differences.

In order to test the influence of language and working memory on children's transcoding performance, three regression analyses were performed. The predictors (phonological working memory, visuospatial working memory, executive working memory, language, IQ, age, and the interactions between language and the three working-memory components) were the same for the three regression analyses.

First, a binary logistic regression analysis was conducted with *type of transcoder* (less- vs. more-skilled) as dependent variable (A in Table 4). Executive working memory was the only significant predictor. Children with higher executive working-memory capacities had more chance to be more-skilled transcoders. A test of the final model (with executive working memory as a predictor) vs. the null model (with intercept only) was statistically significant, χ^2^ (9, *n* = 40) = 14.58 (*p* = 0.05). The final model was able to correctly classify 68% of all children as being less- or more-skilled transcoders. Although digit span forward (a phonological working-memory task) differed between less- and more-skilled transcoders (Table 2), phonological working memory was not significantly predictive of type of transcoder (Table 4), probably because executive working memory accounted for most of the variance shared between these two working-memory components.

**Table 4:** **Standardized beta values of the three regression analyses**.

	**A (transcoder type)**	**B (transcoding errors)**	**C (inversion errors)**
IQ	0.07	−0.04	0.00
Age	0.04	−0.16	0.03
Language	0.00	−0.55	0.51^**^
Visuospatial	−0.55	−2.73	0.09
Phonological	0.62	−3.95^*^	−0.38
Executive	1.67^**^	1.52	−0.67
Language × visuospatial	−0.07	−2.87	0.12
Language × phonological	−0.04	1.23	−0.54
Language × executive	0.35	−0.93	−0.19

* p < .10,

** p < .05, (n = 40).

Second, a linear logistic regression analysis on the *number of transcoding errors* in less-skilled transcoders^3^ (B in Table 4) shows that only phonological working memory tended to be a significant predictor (*p* = 0.06). Less-skilled transcoders with lower phonological working-memory scores made more transcoding errors (*R* = 0.71, adjusted *R*^2^ = 0.68).

Finally, in a linear logistic regression analysis on the *number of inversion errors* (in all children), language was the only significant predictor (C in Table 4). Dutch-speaking children made more inversion errors than French-speaking children (*R* = 0.76, adjusted *R*^2^ = 0.55).

### Role of semantics

In order to test whether children use a semantic or asemantic route when transcoding numbers, we explored the presence of problem size effects (cf. Van Loosbroek et al., 2009). Because the number of errors on 1-digit and 2-digit problems was very small, only 3-digit problems were included in this analysis. A median split was performed on 3-digit problems, dividing them in small problems (*M* = 251) and large problems (*M* = 742) with an equal number of transcoding rules [3.7 and 3.9, respectively, *t*_(38)_ = −1.39, *p* = 0.17]. Less-skilled transcoders made significantly more transcoding errors on large 3-digit numbers than on small 3-digit numbers, *t*_(39)_ = 26.19 (*p* < 0.001), whereas there was no such difference in more-skilled transcoders, *t*_(39)_ = 1.42 (*p* = 0.26). Hence, less-skilled transcoders use a semantic route but more-skilled transcoders use an asemantic route.

### Mathematics achievement

We compared the mathematics grades of the Dutch-speaking less- and more-skilled transcoders in our study. Mathematics grades (average grade in % for maths of the present school year) of the Dutch-speaking children were provided by the schools. Unfortunately, it was not possible to get mathematics grades for the French-speaking children due to reasons of data protection. The more-skilled transcoders achieved significantly higher math scores (89%) than did the less-skilled transcoders (75%), *t*_(18)_ = 3.15 (*p* < 0.01).

## Discussion

We observed an equal number of transcoding errors in Dutch- and French-speaking children. Transcoding errors were more frequent on 3-digit than on 2-digit numbers, indicative of a role of working memory. Regression analyses confirmed that working memory played a significant role in number transcoding. The executive working memory component predicted whether children were less- or more-skilled transcoders. Interestingly, Dutch- and French-speaking children relied on executive resources to a similar degree (the executive working memory × language predictor was not significant). Regarding phonological working memory, we observed that less-skilled transcoders scored lower on the phonological working memory tasks compared to more-skilled transcoders. For the less-skilled transcoders phonological working memory turned out to be predictive of the number of transcoding errors (but the error rate of more-skilled transcoders was too low to test this in more-skilled transcoders). Visuospatial working memory was not predictive. The number of inversion errors in Dutch-speaking children was significantly higher than in French-speaking children. The regression analyses showed that language was the only significant predictor of inversion errors. Thus, although working memory plays an important role in transcoding in general it does not play a specific role in the application of the inversion principle.

### Language

Writing numbers to dictation is a task that adults perform rather faultlessly. Children, in contrast, experience many difficulties in this task (see Table 1). Second graders make fewer transcoding errors than do first graders, and third graders' transcoding performance is near to perfection. The number of transcoding errors did not differ between Dutch- and French-speaking children, indicating that the inversion property, specific for the Dutch number language, had no detrimental effect on children's general transcoding abilities. This is in contrast with the claim made by Pixner et al. (2011), who argued that the inversion property leads to a general increase in transcoding errors. However, in both our and Pixner et al.'s data, the number of non-inversion errors was actually *smaller* in the inversed number language than in the non-inversed number language, providing evidence against the claim that the inversion property would affect children's general transcoding abilities. It is clear that further research is needed into the occurrence of non-inversion errors. Which non-inversion errors are made, and are they more or less frequent in inverted number languages? In our data, for example, we noticed that French-speaking children made about 10% errors on numbers with 80, probably because of the complex French number word “*quatre-vingt*” [literally “*four-(times)-twenty*”].

Similar to transcoding errors, the number of inversion errors in the pretest decreased across age, with fewer inversion errors in second than in first graders, and no inversion errors in third graders. Dissimilar to transcoding errors, is that the number of inversion errors differed across Dutch- and French-speaking children. Dutch-speaking children made significantly more inversion errors than did French-speaking children. In fact, about 20% of the Dutch-speaking children's errors were inversion errors (see Figure 1). The inversion property of the Dutch number language thus results in committing specific errors (inversion errors) reflecting erroneous processing of the inversion rule. Regression analyses confirmed that the number of inversion errors was significantly predicted by a child's number language. These findings corroborate earlier findings (Nuerk et al., 2005; Krinzinger et al., 2011; Pixner et al., 2011; Simmons et al., 2011) by showing that a child's number language strongly influences its transcoding performance. Since in our study both Dutch- and French-speaking children attended Belgian schools, we can conclude that the language effects were truly linguistic, and could not be attributed to differential math curricula (see also Krinzinger et al., 2011).

Interestingly, inversion errors were not predicted by the interaction between language and any of the working-memory components. This indicates that children speaking an inversed number language rely as heavily on their working memory as do children speaking a non-inversed number language. Working memory did play a significant role in transcoding though (albeit the same role in inversed as in non-inversed languages), as discussed below.

### Working memory

Children made significantly more errors on 3-digit than on 2-digit numbers, as was predicted by the ADAPT model of Barrouillet et al. (2004). According to this model, each part of the verbal number word is sent to working memory for storage and processing, which explains why number transcoding is harder as the number of digits increases.

Regression analyses were performed in order to test which working-memory components uniquely predicted children's transcoding performance. Executive working memory significantly predicted children's transcoding skill (i.e., less- vs. more skilled). Children with more executive resources had more chance to be labeled as more-skilled transcoders. The significant role of the central executive is in agreement with Camos (2008), who observed that low-span children transcoded less efficiently than did high-span children, and with Zuber et al. (2009) and Pixner et al. (2011), who observed that executive working memory predicted children's number of transcoding errors. Transcoding verbal number words to Arabic symbols may rely on executive working memory (cf. attentional resources) for a high amount of processing steps, such as retrieving the respective Arabic number symbols from long-term memory, executing transcoding rules, resisting interference from incorrect number representations (e.g., hearing “*fifty*” but writing 15), and coordinating the retention of the partially completed digit chain while applying subsequent transcoding rules. The significant effect of executive working memory is in disagreement with Simmons et al. (2011), who—in contrast to their own expectation—observed no significant role for the central executive^4^. However, Simmons and colleagues note that the central executive not explaining unique variance does not exclude a role for this working-memory component. They contribute their null effect to the fact that the variance explained by the executive component covaried with the variance explained by the visuospatial component. In sum, our study confirms the conclusion, drawn by most recent studies as well, that executive resources play a significant role in children's number transcoding.

Our results may suggest that phonological working memory plays a role in transcoding as well, albeit only in less-skilled transcoders. Less-skilled transcoders with fewer phonological resources made more transcoding errors. Although more research is necessary, this is an important result, especially given the discrepancy between the predictions of the ADAPT model, according to which phonological resources are crucial in number transcoding (Barrouillet et al., 2004), and the absence of empirical evidence for this claim (Zuber et al., 2009; Pixner et al., 2011; Simmons et al., 2011). Phonological resources may be needed for the maintenance of intermediary information, such as the dictated verbal number word, the retrieved Arabic digits, and the chain under construction. Because less-skilled transcoders (who have fewer executive resources available) transcode less efficiently, they probably have more information to be stored simultaneously, increasing their reliance on phonological resources.

We did not observe a role of visuospatial working memory in number transcoding. This is in accordance with the results of Pixner et al. (2011), who neither observed a role for this working memory component in children's number transcoding. Our results are, however, in contrast with some other studies, where visuospatial working memory did predict children's transcoding errors (Simmons et al., 2011) or non-inversion errors (Zuber et al., 2009). Whether or not children retain a visuospatial representation of the digit chain when they are transcribing number words, is a question for further research. We propose that the answer to this question depends on several factors, such as the age of the children (with younger children relying exclusively on spatial coding and dual (visuospatial + phonological) coding arising around 8 years; Palmer, 2000; Pickering and Gathercole, 2001) and the type of errors (with visuospatial processes being more important in non-inversion errors, cf. Zuber et al., 2009).

Finally, it is important to note that none of the language × working memory variables did predict the number of transcoding errors. This indicates that the role of the different working-memory components is similar in children speaking inversed and non-inversed languages. This is somewhat surprising; given that the inversion property requires extra steps (such as memorizing and manipulating the sequence of number words), one might have expected that executive resources would be particularly related to inversion errors. As this study was the first one testing the role of working memory in children speaking inversed and non-inversed number languages, we hope that further studies will continue on this line of research. It might be interesting to contrast inversed number languages with completely transparent number languages (e.g., Chinese, where 264 is “*two-hundred*-*six-ten-four*”).

### Implications

Given the vast number of inversion errors, a first implication of our study is a theoretical one. As neither semantic nor asemantic transcoding models do in their current form account for inversion errors, this urges for a revision of these models. Note that the inversion principle does not only exist in Dutch, but also in other languages such as Arabic, Danish, Czech, German, Maltese, Malagasy, and Norwegian (Comrie, 2005). Hence, if transcoding models aim to be generally applicable, they have to add additional operators (such as inversion rules) in order to account for the errors observed in these languages. Connectionist modeling is informative here. Starting from a specific model like the one of Verguts and Fias (2006), it would of course be necessary to formally check if different network parameters, architectural constraints or training schemes would be necessary for a connectionist network to learn syntactic rules that include inversion. But, given that inversion by itself does not imply a serious rise of computational complexity or a change of underlying computational principle, there is no reason to expect that models of the type of Verguts and Fias (2006) are not capable of learning inversion. Hence, it can be reasonably expected that non-semantic models, equipped with a system for representing syntactic rules, can explain the specificities of inversion-related behavior.

A second theoretical implication concerns the role of working memory in number transcoding. The ADAPT model (Barrouillet et al., 2004) is the only transcoding model explicitly incorporating a role for working memory. As our and others' data show that working memory plays a significant role in children's number transcoding (Camos, 2008; Zuber et al., 2009; Pixner et al., 2011; Simmons et al., 2011), *all* transcoding models should actually pay attention to this influencing variable.

A last theoretical consideration concerns the dissociation between semantic and asemantic models: which type of models accounts best for the data? Based on recent evidence suggesting that route selection may depend on children's mathematical skill (with semantic routes being more frequently used in mathematically disabled children; Van Loosbroek et al., 2009), we tested if this was also true in typically developing children. According to our data, it looked as if less-skilled transcoders used a semantic route whereas more-skilled transcoders used an asemantic route. Hence, the selection between semantic and asemantic routes seems to depend on children's mathematical skill rather than being an all-or-none phenomenon. The observation of number size effects in the performance of mathematically disabled children might in principle reflect non-semantic effects of familiarity or of exposure rather than semantic effects emanating from the use of a semantic route. However, such an explanation is less likely to explain the number magnitude effect observed in the present study. Our participants were skilled readers and they had a highly similar educational curriculum. Therefore we can assume that they have had the same level of exposure to numbers. It would be interesting to further test this hypothesis not only as regards to error rates (as in the present study) but also as regards planning time (as in Van Loosbroek et al., 2009, who used graphic tablets that recorded children's pen trajectories). It would also be interesting to investigate the developmental trajectory to see whether the semantic transcoding established in less skilled readers presents as an intermediate stage in skilled transcoders at less skilled stages of development.

It should be noted that for cost-efficiency reasons, this study was conducted with only a small subset of less-and more skilled transcoders. Following Preacher et al. (2005), one should realize that this may come at a cost. First, it may lower power, especially if the two subsets are treated as a dichotomous variable. We do treat transcoding skill as a dichotomous variable in some analyses, like the t-tests and the binary regression. Yet, the fact that we also used regression analyses with number of errors as continuous dependent variable at least partially protects us against this problem. Second, using extreme groups leads to the inability to derive the exact nature of any non-linear relationship between the group variable and the variables under study. Because we consider this study to be exploratory and because existing theoretical models are not developed to such an extent that any claim is made about a precise relationship, we do not consider this to be a threat. Finally, one must be cautious in generalizing the results to all school children, given the small number of participants.

Besides the theoretical implications, there are also practical implications. Recent evidence suggests that number transcoding may be a very important precursor of mathematical skill. In a longitudinal study, Moeller et al. (2011) showed that the number of inversion errors made in first grade reliably predicted children's addition performance in third grade. The number of inversion errors in first grade was also the only reliable predictor of mathematics grades in third grade. In the present study, we observed (for Dutch-speaking children only as mathematics grades were not available for French-speaking children) that the more-skilled transcoders achieved significantly higher math scores (89%) than did the less-skilled transcoders (75%), indicating that there is a meaningful relationship between the very basic skill of number transcoding and the more complex skill of mathematical problem solving. This implies that educators should give adequate attention to the mastery of the place-value system of the Arabic number system. Indeed, as argued by Moeller et al. (2011), transcoding errors indicate that children do not master the correspondence between verbal number words and the place-value structure for Arabic digits.

Given that transcoding errors can be an indication of arithmetical disabilities (e.g., Gross-Tsur et al., 1996; Hanich et al., 2001; Van Loosbroek et al., 2009), place-value understanding might also be a crucial factor in explaining mathematical difficulties. As such, we believe that further research should focus on number transcoding as an early precursor of later mathematical skill, and as a possible indicator of arithmetical disabilities.

## Author note

Support for this research was provided by the Research Foundation Flanders (FWO Flanders) with a postdoctoral fellowship to Ineke Imbo.

### Conflict of interest statement

The authors declare that the research was conducted in the absence of any commercial or financial relationships that could be construed as a potential conflict of interest.

## Footnotes

^1^In Czech, the easier, non-inverted form of number words (e.g., 25 verbalized as “dvadsetpät” [twenty-five]) is taught at school, whereas the inverted form (e.g., 25 verbalized as “pätadvadset” [five-and-twenty]) is commonly used in daily life. There is thus still a confound between cultural and educational influences, on the one hand, and number language, on the other.

^2^In contrast to the French number language in France, where 70 is “soixante-dix” (sixty-ten) and 90 is “quatre-vingt-dix” (four-twenty-ten), the Walloons say “septante” and “nonante,” respectively. All other numbers are pronounced similarly by Walloons and French children.

^3^More-skilled transcoders were not included in this analysis because their number of transcoding errors was too small.

^4^Another peculiarity in Simmons et al. (2011) method is that the numbers were presented with increasing problem size. The children could thus reliably guess (a) the problem size of the next number, and (b) the number of digits in the next number, which might have decreased their need for executive control.
