# Supplementary material for: Sixty-four or four-and-sixty? The influence of language and working memory on children's number transcoding
Source: Front Psychol. 2014 Apr 11;5:313. doi: 10.3389/fpsyg.2014.00313 (PMC3990049; doi:10.3389/fpsyg.2014.00313)
Supplement: Supplementary file 1 [file Presentation1.PDF]

Appendix 1

Amount of dictated numbers in as a function of the categories and transcoding rules of the ADAPT model (Barrouillet et al., 2004).

Category	P1	P2	P3	P4	Total	Example	Amount
U	1	0	0	1	2	4	5
P	1	0	0	1	2	12	2
D	1	0	0	1	2	30	2
DU	1(2)	0	0	1	2(3)	39	16
H	0	1	0	1	2	100	1
HU	1	1	0	2	4	108	2
HP	1	1	0	1	3	117	2
HD	1	1	0	1	3	150	2
HDU	1(2)	1	0	1	3(4)	186	2
UH	1	1	0	1	3	400	2
UHU	2	1	0	2	5	409	2
UHP	2	1	0	1	4	614	2
UHD	2	1	0	1	4	350	3
UHDU	2(3)	1	0	1	4(5)	845	22

U = Unit, P = Particular, D = Decade, H = Hundred. P1 rules are responsible for retrieval from long-term memory, P2 and P3 rules are responsible for managing hundreds and thousands, P4 rules are stop rules. Total = number of transcoding rules required when the DU's are retrieved from long-term memory (resp. when the DU's are algorithmically transcoded).
